# Increased AID Results in Mutations at the CRLF2 Locus Implicated in Latin American ALL Health Disparities

**DOI:** 10.21203/rs.3.rs-3332673/v1

**Published:** 2023-09-11

**Authors:** Nicholas Pannunzio, Valeria Rangel, Jason Sterrenberg, Aya Garawi, Vyanka Mezcord, Melissa Folkerts, Sabrina Caulderon, Jinglong Wang, Eli Soyfer, Oliver Eng, Jennifer Valerin, Sora Tanjasiri, Fabiola Quintero-Rivera, Selma Masri, Marcus Seldin, Richard Frock, Angela Fleischman

**Affiliations:** UC Irvine; UC Irvine; UC Irvine; UC Irvine; California State University Fullerton; UC Irvine; UC Irvine; Stanford University; UC Irvine; UC Irvine; UC Irvine; UC Irvine; UC Irvine; University of California, Irvine; University of California, Irvine; Stanford University; University of California, Irvine

## Abstract

Activation-induced cytidine deaminase (AID) is a B cell-specific base editor required during class switch recombination and somatic hypermutation for B cell maturation and antibody diversification. However, it has also been implicated as a factor in the etiology of several B cell malignancies. Evaluating the AID-induced mutation load in patients at-risk for certain types of blood cancers is critical in assessing disease severity and treatment options. Here, we have developed a digital PCR (dPCR) assay that allows us to track the mutational landscape resulting from AID modification or DNA double-strand break (DSB) formation and repair at sites known to be prone to DSBs. Implementation of this new assay showed that increased AID levels in immature B cells increases genome instability at loci linked to translocation formation. This included the *CRLF2* locus that is often involved in chromosomal translocations associated with a subtype of acute lymphoblastic leukemia (ALL) that disproportionately affects Latin Americans (LAs). To support this LA-specific identification of AID mutation signatures, we characterized DNA from immature B cells isolated from the bone marrow of ALL patients. Our ability to detect and quantify these mutation signatures will potentiate future risk identification, early detection of cancers, and reduction of associated cancer health disparities.

## Introduction

Diversification of the immunoglobulin heavy chain *(IGH)* locus in B cells is a form of physiological genome editing that was been conserved over 500 million years of evolution.^1,2^ As B cells mature, DNA double-strand breaks (DSBs) are generated at two different points by two distinct mechanisms to modify the region of the *IGH* gene that encodes for either the antibody variable region or the antibody constant region.^3^ In pro-B/pre-B cells, the recombination activating gene (RAG) complex is expressed to generate DSBs at variable (V), diversity (D), and joining (J) gene segments during V(D)J recombination.^4^ In mature B cells, activation-induced cytidine deaminase (AID) is expressed to initiate the processes of class-switch recombination (CSR) and somatic hypermutation (SHM).^5^

AID evolved to be an integral part of the adaptive immune system,^6^ but its expression must be tightly sequestered to mature B cells as off-target deamination events have the potential to result in mutations genome-wide. The consequences of aberrant AID expression and off-targeting are evident in the etiology of several B cell cancers.^3,7^ Further, that AID is B cell specific, and B cell cancers account for approximately 80–90% of hematopoietic malignancies,^3,8^ is a further indicator of the dangers of abnormal AID activity. Sequestering the RAG-induced DSBs of V(D)J recombination to pre-B cells and the AID-induced DSBs of CSR to mature B cells helps maintain genome stability. However, AID can be transiently expressed in pre-B cells concurrent with RAG expression when pre-B cells are stimulated by cytokines.^9^ Indeed, examination of the various chromosomal translocations that occur in many lymphoid malignancies show that one DSB was RAG-induced at the *IGH* locus adjacent to a D or J cassette while the other DSB locus shows evidence of AID activity,^10^ indicating the most likely mechanism for oncogenesis in a subset of cancers is AID expression in pre-B cells.^11^

Understanding both why some RAG-induced DSBs in pre-B cells fail to be efficiently repaired by nonhomologous end-joining (NHEJ) and what leads to AID activity in pre-B cells will allow us to better predict populations susceptible to certain blood cancers and discern the etiology of these malignancies. Two loci that show mutations consistent with AID activity in pre-B cells are *BCL2* and *CRLF2*. ^10,12,13^ Unlike random DSB sites that can span tens or hundreds of kilobases, these AID-induced DSBs are focal with many occurring within 20–600 bp regions that we refer to as AID break clusters (ABCs).

A critically understudied area in genome instability associated with diseases are cancer health disparities where one racial or ethnic group shows higher incidence and poorer outcomes of certain cancer types.^14^ For example, Philadelphia chromosome-like acute lymphoblastic leukemia (Ph-Like ALL) has a higher cancer incidence in Latin Americans (LAs).^15–17^ Ph-like ALL shares several characteristics with Philadelphia-positive (Ph^+^) ALL regarding the gene expression profile, yet lacks the eponymous t(9;22) *BCR-ABL1* translocation.^18^ LAs specifically have a higher likelihood of developing Ph-like ALL and respond less favorably to current standard treatments.^15,19^ Genome rearrangements and mutations leading to Ph-like ALL are highly diverse, yet LAs are more likely to have Ph-like ALL characterized by a translocation between *IGH* and the ABC upstream of the *CRLF2* locus.^15,19^ This is in contrast to Europeans and non-Latin Americans that more often have an interstitial deletion between *CRLF2* and *P2RY8*.^20^ While some risk alleles have been identified that link LAs to higher Ph-like ALL risk,^9^ no molecular mechanism has been established to understand why *CRLF2-IGH* formation is more prevalent in this population.

Here, using both human cell lines and patient samples we identify an increase in insertion and deletion events (indels) at two ABCs, the *CRLF2* ABC linked to LAs with Ph-like ALL and the non-LA linked *BCL2* ABC linked to follicular lymphoma. In many cases, these indels are the “informational scars” left behind by NHEJ DSB repair due to processing factors that remove or add nucleotides prior to ligation.^14,21^ To deeply characterize these genetic changes, we developed a digital PCR (dPCR) assay to detect and quantify changes at the CpG sites in ABCs targeted by AID. We show that increased AID expression correlates with an increase in indels at these ABCs. Strikingly, these indels are also detected in ALL patient samples but not samples from healthy donors, indicating that dPCR can provide a novel diagnostic for early detection of patients with aberrant AID expression that would increase cancer risk.

## Materials and methods

### Cell lines and culture conditions

Nalm6 and Reh cell lines were purchased from the American Type Culture Collection (ATCC, Manassas, Virginia) and grown in RPMI-1640 supplemented with 10% FBS, 1% Penicillin/Streptomycin, 1% Glutamax (Gibco), and 50 μM b-mercaptoethanol. Cell lines with the transduced AID doxycycline (dox)-inducible cassette were grown under G418 selection (1 mg/mL of G418). Cells with constitutive Cas9 expression were grown in complete media further supplemented with 0.75 μg/mL of Puromycin. All cell lines stated above were grown at 37°C and 5% CO_2_.

### Amplicon Sequencing

DNA regions were amplified from Nalm6, Reh, and patient gDNA using primers with partial Illumina adapter sequences (**Supplemental Table 1**). PCR reactions were performed using Platinum Taq DNA Polymerase (Invitrogen). Amplified DNA fragments were purified using HighPrep PCR clean-up magnetic beads (MAGBIO, Gaithersburg, MA) at 1.8x concentration. SNP/INDEL detection analysis was performed by Genewiz’s Amplicon E-Z Next Generation Sequencing (Azenta Life Science, South Plainfield, NJ).

### Vector construction

Single guide RNAs (sgRNAs) targeting ABCs were designed using Benchling. Synthesized DNA oligos (Integrated DNA Technologies (IDT), Coralville, IA) were ligated into the *Bbs*I sites of pSPgRNA^22^. Gateway cloning was used to generate pINDUCER-AID using the Invitrogen Clonase II kit to integrate AID into pINDUCER20^23^. Nucleofections were performed using the Amaxa Biosystems Nucleofector II with program T-001.

### Construction of cells with dox-inducible AID or constitutive Cas9 cassettes

Virions with pINDUCER-AID were generated using a 3rd generation lentiviral system in HEK293T cells.^24^ For constitutive Cas9, the lentiCRISPRv2^25^ vector was used. Nalm6 and Reh cell lines with the dox-inducible AID cassette (hereon referred to as Nalm6-AID and Reh-AID) were generated by transduction and selection in complete media supplemented with 1 mg/mL of G418. A similar process was done using virions that incorporate the Cas9 expression cassette and create Nalm6-Cas9 with selection done using 0.75 μg/mL of puromycin.

### RNA isolation and qPCR

Total RNA was extracted from cells using TRIzol (Thermo Fisher Scientific) as described by the manufacturer’s instructions. cDNA was generated using the Maxima H Minus cDNA Synthesis Master Mix (Thermo Fisher Scientific) using 1 μg of total RNA according to the manufacturer’s instructions. The cDNA was then used as template in quantitative real-time PCR using a SYBR Green Master Mix (Applied Biosystems). Gene expression was normalized to 18S ribosomal RNA. Primer sequences used for gene expression analysis are listed in Supplemental Table 1.

### RNA-seq Analysis

Poly-A enriched RNA was isolated from patient samples stored at −80°C in TRIzol for library preparation and 150 bp paired-end sequencing was performed by Novogene (Beijing, China). FASTQ files for RNA-seq have been deposited in Sequence Read Archive (SRA, SPR# accession pending). FASTQ files were inspected for base quality scores using FASTQC and aligned to the human genome (GRCh38, bioproject PRJNA31257) using STAR aligner.^26^ From aligned BAM files, PCR duplicates were removed using MarkDuplicates in gatk.^27^ Next, counts matrices were assembled from BAM files in using the summarizeOverlaps function from the GenomicAlignments package.^28^ Counts matrices were then filtered for genes with a rowSum of 10 counts across all samples then differential expression was performed using DESeq2.^29^

### Protein isolation and Western blotting

Protein lysates containing an equal amount of protein were loaded and resolved on an SDS–PAGE gel and transferred to a nitrocellulose membrane. For imaging, membranes were treated with Immobilon Western Chemiluminescent Substrate (Millipore). Chemiluminescent images were obtained using the BioRad ChemiDoc System. Antibodies used for the Western blot were: AID (L7E7) (Cell Signaling, 4975), FLAG M2 (Sigma-Aldrich, A2220), and p84 (GeneTex, GTX70220).

### dPCR

dPCR samples were prepared as described previously.^30^ Briefly, 9 μL reactions contained 1x Absolute Q DNA dPCR Master Mix (Thermo Fisher Scientific), 900 nM of each forward and reverse amplicon primer, 250 nM of FAM, TAMRA, Cy5 and/or SUN/HEX TaqMan probes, and 10–20 ng of gDNA prepared by phenol:chloroform extraction and ethanol precipitation. Primer and probe sequences used for dPCR drop-off assays are listed in Supplementary Table 1. Quantstudio software uses Poisson distribution that allows for quantification of each amplified product in copies/μL based on the fluorescent signal in each microchamber.

### High-Throughput Rejoin and Genome-Wide Translocation Sequencing (HTGTS-JoinT-seq)

HTGTS was performed using the linear amplification-mediated (LAM) platform^31^ with modifications as described^31^ to quantify both single DSB rejoining as well as translocation to other DSBs (JoinT-seq). Briefly, 10 μg of isolated gDNA was used to prepare amplicons for Illumina Nova-seq 150 bp paired end sequencing. Sequence reads were aligned to the hg38 genome build and normalized to 2,914,360 and 793,219 each for IGHM-6 and IGHM-1 bait analyses, respectively. Hotspot determination used MACS2 with an FDR-adjusted P-value cutoff of 10^− 9^ as described.^32^

### Patient Samples

Patient samples were obtained through two UCI IRB approved protocols. Samples were obtained from bone marrow or blood (if bone marrow was not available and blasts were > 50% in peripheral blood). De-identified healthy donor blood samples were provided by the UCI Institute for Clinical and Translational Science. Mononuclear cells were obtained by ficol gradient centrifugation followed by a brief red blood cell lysis step in ammonium chloride potassium (ACK) buffer. Cells were used fresh or thawed from frozen cells preserved in 90% Fetal Bovine Serum (FBS) + 10% DMSO.

## Results

### Amplicon sequencing (AmpSeq) of ABCs shows increased indels in human B cells

**Figure 1:**
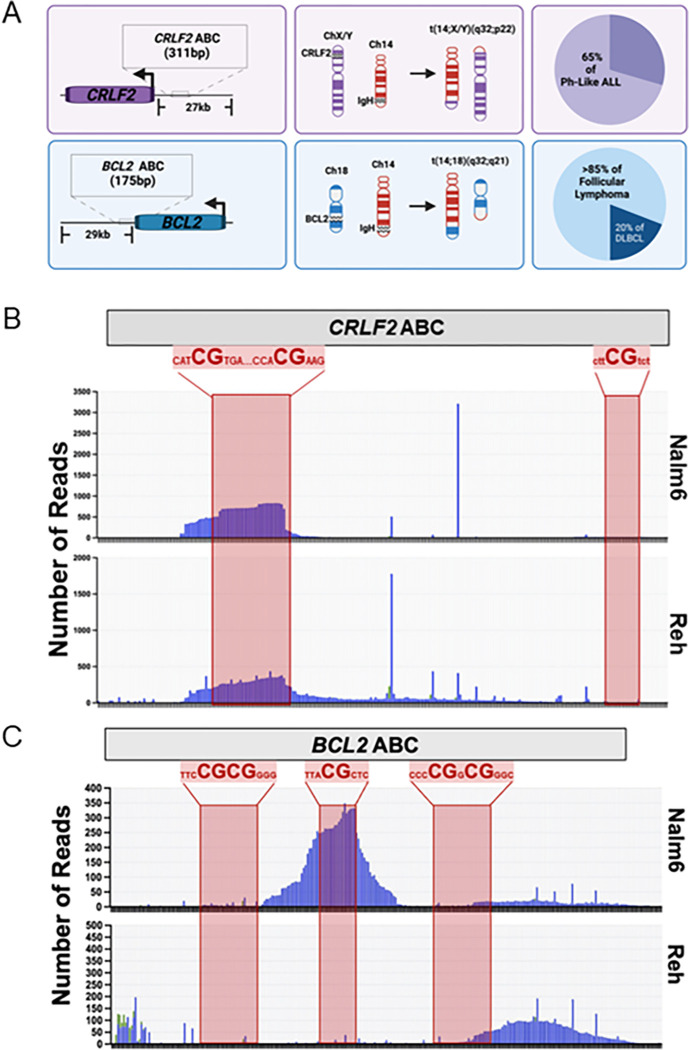
AID Break Clusters (ABCs) associated with recurrent translocations in B cell cancers show evidence of genome instability. **(A)** Diagram of *CRLF2* and *BCL2* ABC sites and their locations relative to their corresponding gene. The oncogenic translocations formed between each of the two ABC sites and *IGH* are represented in the middle column, where pathological (AID) and physiological (RAG) double-stranded breaks are portrayed by open gaps in the chromosomes. The percentage of the B cell cancer subtypes associated with the oncogenic translocations are shown in the last column with data taken from ^3,19,45^. Insertion and deletion (indel) events detected by AmpSeq at **(B)** *CRLF2* and **(C)** *BCL2* ABC sites in Nalm6 (top panel) and Reh (bottom panel) cells. CpG sites spanning each of the three ABC sites are represented by the partial nucleotide sequence shown above the red highlighted regions overlaying the amplicon sequencing data (see **Supplemental Figure 1** for detailed sequence information). Each bar represents changes at a single base pair with deletions (blue) more dominant than insertions (green).

Of the known ABCs associated with B cell malignancies,^3^ the major focus here is on the ABC upstream of the *CRLF2* gene.^13^*CRLF2* is located on a pseudoautosomal region (PAR) shared by both the X and Y chromosomes.^33^ We also include data on the ABC in the 3’ UTR of the *BCL2* gene that is also called the *BCL2* Major Breakpoint Region (MBR) (Fig. 1A) to demonstrate that more than one ABC site can be targeted even though only a DSB at one leads to an oncogenic translocation with *IGH*.^10^

According to the mapping of chromosomal translocation junctions in human patients, a DSB that results in pathogenic rearrangements involving *CRLF2* can occur anywhere in the 27 kb region upstream of the gene, yet two specific DSB clusters are of note.^13^ Approximately 4.5 kb upstream of *CRLF2* is a cluster that shows the CAC/CACA motif associated with being a cryptic recombination signal sequence (RSS) recognized by the RAG complex. Off-target RAG cutting at this site leads to an intra-chromosomal deletion that puts the *CRLF2* gene under control of the *P2RY8* promoter, leading to its overexpression.^13,34^ The other site is 16 kb upstream of *CRLF2* and contains no cryptic RSS sites, but does have DSBs at or near a CpG, which is a hallmark of ABCs since AID has an affinity to deaminate cytosines within RCG sequences (WGCW>>>WRC > > RCG).^35–37^ For rearrangements involving ABCs, it has been shown that a translocation is 30% more likely to occur directly at a CpG dinucleotide and 70% more likely to occur within 8 bp of a CpG dinucleotide.^10^

AID requires single-stranded DNA (ssDNA) as a substrate for deamination.^35^ While AID expression in the human pre-B cell lines Nalm6 and Reh is below the limit of detection,^38^ the ssDNA generated at these sites would still be vulnerable to damage. Thus, we were curious if ABCs show an inherent propensity for damage. This was tested using amplicon sequencing (AmpSeq). The detailed sequence for the *CRLF2* region with the ABC is shown in Supplemental Fig. 1A. Strikingly, as with the reported patient data,^10,13^ there is a peak of deletions near the CpGs in the *CRLF2* ABC in both Nalm6 and Reh cell lines (Fig. 1B). These deletions likely represent information scars indicating that these are sites of DSB formation and NHEJ repair, which is prone to creating indels. In Nalm6 cells, indels appear very focal and are highest at the first two CpG sites. While indels are also highly clustered at this site in Reh cells, we found that the indels appear more spread throughout the ABC. In both cell lines the downstream CpG does not appear to accumulate indels.

Similarly, using AmpSeq at the *BCL2* ABC (**Supplemental Fig. 1B**), we also see indels, yet observe a notably different pattern for each cell line (Fig. 1C). It has been reported that DSBs cluster at the three regions carrying CpG sites in patients.^10^ In Nalm6 cells, we measure a major peak centered on the middle CpG site, but no indels at the flanking CpG sites. In contrast, while there is still a peak in Reh cells, it is downstream of the three CpG sites. This may be indicative of either this region being less accessible to enzymes that drive formation of these indels or that DSBs ends are processed or resected differently once formed. Importantly, that indels accumulate in the vicinity of CpGs mapped at both *CRLF2* and *BCL2* to correlate with the regions where DSBs involved in chromosomal translocations have been mapped in human patients^10,13^ shows these regions have an innate instability consistent with results studying their cytosine content.^39^

### CRISPR/Cas9 demonstrates ability of digital PCR (dPCR) to detect indels at ABCs

**Figure 2:**
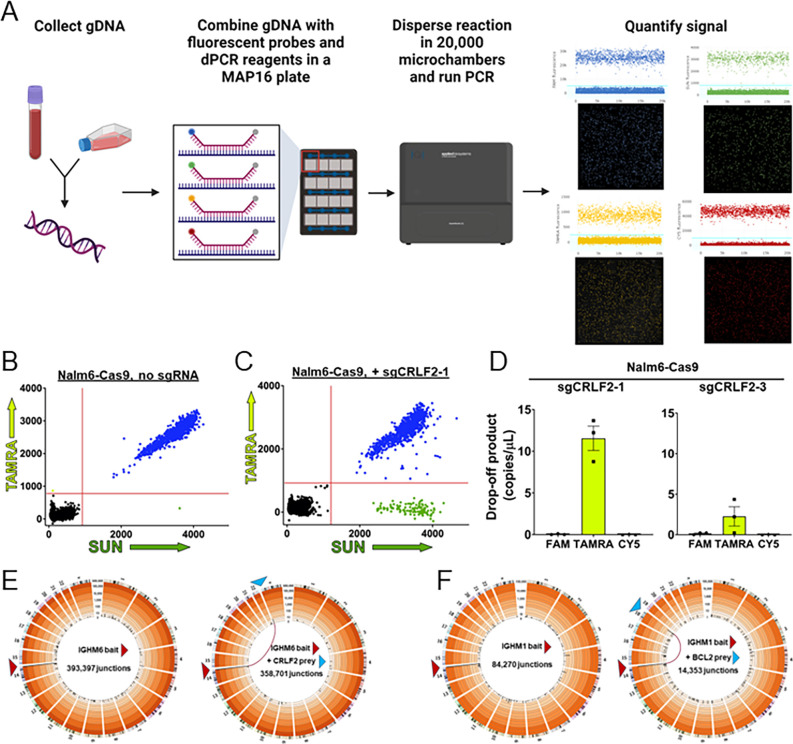
Digital PCR (dPCR) can detect and quantify indels following DSB formation and repair at the *CRLF2* ABC. **(A)** Workflow for dPCR assay using the Applied Biosystems Absolute-Q. Harvested gDNA is combined with amplicon primers, TaqMan probes, and master mix then loaded onto a MAP16 plate where the reaction is partitioned into over 20,000 microchambers. A representative experiment showing quantification of fluorescent signal from 4 channels indicates the detected signal from each microchamber. **(B)** dPCR results in cells with no Cas9 cutting. Results are shown as a 2D scatterplot with the fluorescent signal from each of 20,000 individual microchambers indicated. Colors represent SUN alone (green) TAMRA alone (yellow/green), SUN+TAMRA (blue), or microchambers with no signal (black). While only TAMRA and SUN are shown here, fluorescent signals from all four probes are gathered simultaneously. **(C)** dPCR results as a 2D scatter plot following Cas9 cutting after transfection of a vector expressing sgCRLF2–1 in a Nalm6 cell line with an integrated Cas9 expression cassette (Nalm6-Cas9). The increase in SUN alone signal indicates a significant drop-off of the TAMRA probe binding the Cas9 cut site (Supplemental Figure 1A). **(D)** Quantification of drop-off product in copies/mL as determined by detection of amplicons that are bound by the SUN probe, but not the FAM, TAMRA, or Cy5 probes in Nalm6-Cas9 cells following transfection of a vector expressing either sgCRLF2–1 or sgCRLF2–3. Data is plotted as mean values ± SEM from three replicates. (**E**)Circos plots demonstrating breakpoint junctions and/or translocations in Nalm6-Cas9 cells transfected with sgIGH-6 (bait) alone or co-transfected with sgIGH-6 and sgCRLF2–1. (**F**) Circos plots demonstrating breakpoint junctions and/or translocations in Nalm6-Cas9 cells transfected with sgIGH-1 alone or co-transfected with sgIGH-1 and sgBCL2. For E and F, a red arrow indicates the *IGH*bait DSB and a blue arrow indicates the *CRLF2* or *BCL2* prey DSB. Total number of breakpoint junctions measured is indicated. Connecting red lines show the bait/prey translocation formed.

While AmpSeq can provide an analysis of sequence changes occurring at ABCs, we sought an improved method that was more rapid and quantifiable to detect mutations within ABCs and developed a novel dPCR-based assay^40^ for the *CRLF2* and *BCL2* ABCs (**Supplemental Fig. 1A and 1B**). For *CRLF2*, three TaqMan probes bind directly at one of the three CpG sites (FAM, TAMRA, Cy5). These are drop-off probes as DSB formation and repair would alter the sequence, preventing probe binding (*i.e*., it “drops-off”). The SUN reference probe binds where no DSBs have been mapped and acts as a detector for the amplicon and a baseline for the total fluorescent signal produced. Genomic DNA (gDNA) from cells or patient blood can then be collected and subjected to dPCR analysis to determine genome stability at specific loci (Fig. 2A).

We tested the sensitivity of the dPCR assay using a CRISPR/Cas9 system to induce DSBs. Two single-guide RNAs (sgRNAs), sgCRLF2–1 and sgCRLF2–3, were designed to target Cas9 to sites bound by the TAMRA drop-off probe (**Supplemental Fig. 1A**) and were transfected into Nalm6-Cas9 cells (**Supplemental Fig. 2**). dPCR was performed on gDNA from untransfected Nalm6-Cas9 cells or cells transfected with sgCRLF2–1. 2D plots of the dPCR results are shown in Figs. 2B and 2C. No significant accumulation of the drop-off product is measured in untransfected cells as nearly all amplicons are bound by both TAMRA and SUN probes (Fig. 2B). Upon transfection of sgCRLF2–1, there is a dramatic increase in the level of drop-off product (*i.e*., an amplicon where the SUN probe binds but the TAMRA probed does not) (Fig. 2C). Comparing the copies/μL of drop-off product across multiple replicates demonstrates the high degree of consistency between experiments and shows that transfection of sgCRLF2–1 results in approximately 10% of cells showing drop-off of the TAMRA probe, a 1000-fold increase over the baseline levels leading to over 10 copies/μL of drop-off product (Fig. 2D). Interestingly, if we compare this to sgCRLF2–3, which recognizes a site on the opposite DNA strand and was predicted to cut as efficiently as sgCRLF2–1, we measure 5-fold less drop-off.

To determine if Nalm6-Cas9 cells harbor ongoing genome instability at ABC sites or if the sgRNAs used display off-target activity, we used High-Throughput rejoin and Genome-Wide Translocation Sequencing (HTGTS-JoinT-seq).^41^ An sgRNA that recognizes a region of the *IGH* gene that is unrearranged in Nalm6 cells (sgIGH-6, **Supplemental Table 1**) was used to create the bait DSB to map breakpoints genome-wide. Compared to no bait sgRNA controls, which showed very low numbers of random breakpoint junctions (283 and 161 total breakpoint junctions, **Supplemental Fig. 3**), sgIGH-6 transfection resulted in over a 1000-fold increase in junctions (Fig. 2E), but no ABC hotspot translocation events. Recurrent t(X;14) translocation events were only detected after co-transfection with sgCRLF2–1 (Fig. 2E). Similar results were obtained with a sgRNA that targets Cas9 to *BCL2* (Fig. 2F). These results indicate that Nalm6 cells are not undergoing genome destabilizing events at the two ABC sites at levels detectable by joins to the *IGH*-induced DSB.

### dPCR detects increased indels in response to increased AID expression

**Figure 3:**
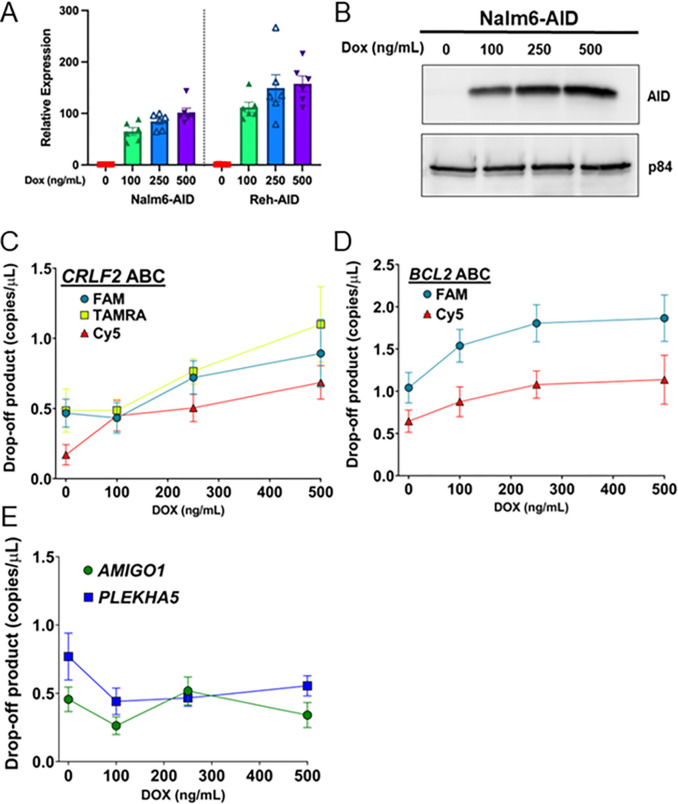
dPCR detects increased indels in response to increased AID expression in human pre-B cell lines. **(A)** Relative expression of the *AICDA* gene encoding AID in Nalm6 and Reh cells with a lentivirally-integrated dox-inducible AID cassette determined by qPCR in cells cultured with indicated amounts of doxycycline. Mean values ± SEM (*n*= 6). **(B)** Western blot showing protein abundance of AID in dox-induced Nalm6-AID cells relative to p84. **(C)** Quantification of dPCR drop-off products for the FAM, TAMRA, and Cy5 probes associated with the *CRLF2* ABC site using gDNA harvested from Nalm6-AID cells induced with indicated concentrations of doxycycline. **(D)** Quantification of dPCR drop-off products for the FAM and Cy5 probes associated with the *BCL2* ABC using gDNA harvested from Nalm6-AID cells induced with the indicated concentrations of doxycycline. **(E)** dPCR of the *AMIGO1* control locus, Chr 1: 109,507,297–109,507,477 (hg38) and the *PLEKHA5* control locus, Chr 12: 19,358–238-19,358,405 (hg38). dPCR results are indicated as mean values ± SEM from 3 biological replicates performed in triplicate.

To directly correlate AID activity with indel formation at ABCs, we integrated a doxycycline (dox) inducible AID expression cassette via lentiviral transduction to generate Nalm6-AID cells. While no protein was detectable with 0 ng/mL of dox, increases in both AID expression (Fig. 3A) and protein levels (Fig. 3B) were evident as dox levels increased. gDNA was prepared from Nalm6-AID cells grown in increasing levels of dox for dPCR analysis of the *CRLF2* ABC (Fig. 3C). With no dox, the drop-off products indicating mutations at the FAM and TAMRA bound sites are elevated while the Cy5 site shows little drop-off. This is very consistent with the AmpSeq data of this region and again demonstrates that the region bound by FAM and TAMRA is inherently unstable while the WGCW at Cy5 is not. Strikingly, with just 100 ng/mL of DOX to increase AID levels, there is a sharp increase in drop-off of the Cy5 probe that continues to rise as more DOX is added showing a clear dose response. Drop-off at the FAM and TAMRA sites also rise as AID levels increase with 250 and 500 ng/mL DOX treatment showing that, on top of the mutations already present, AID can further increase mutations at the *CRLF2* ABC.

A similar dose response correlating increased AID expression with increased drop-off was also measured for the *BCL2* ABC (Fig. 3D). The Cy5 bound site, which showed the most instability via AmpSeq in Nalm6, shows a sharp increase in response to initial DOX treatment while mutations at the FAM site continue to increase in response to increasing DOX levels. This demonstrates that both the *CRLF2* and *BCL2* ABCs are assessable and targeted by AID.

Importantly, we also tested non-ABC loci that harbor CpG sites, *AMIGO1* (chr1: 109,507,297, 5’-CACAATGGGCGTATCA-3’) and *PLEKHA5* (chr12: 19,358,238, 5’-TGACTGTGGAAGAGCA-3’), by dPCR. Neither site shows any increased drop-off as AID levels increase in the cells showing that the RCG and WGCW sites at these control regions do not accumulate mutations (Fig. 3E). While this list is not exhaustive, and we cannot rule out that some non-ABC sites are targeted, the stark difference between these control loci and the ABC sites show that the latter are much more heavily targeted by AID activity. This data supports that ABCs at *CRLF2* and *BCL2* are unique features prone to AID damage, likely related to these sequences being dynamic and able to form transient ssDNA structures. ^39,42^

### Amplicon sequencing of patient samples shows increased indels

**Figure 4:**
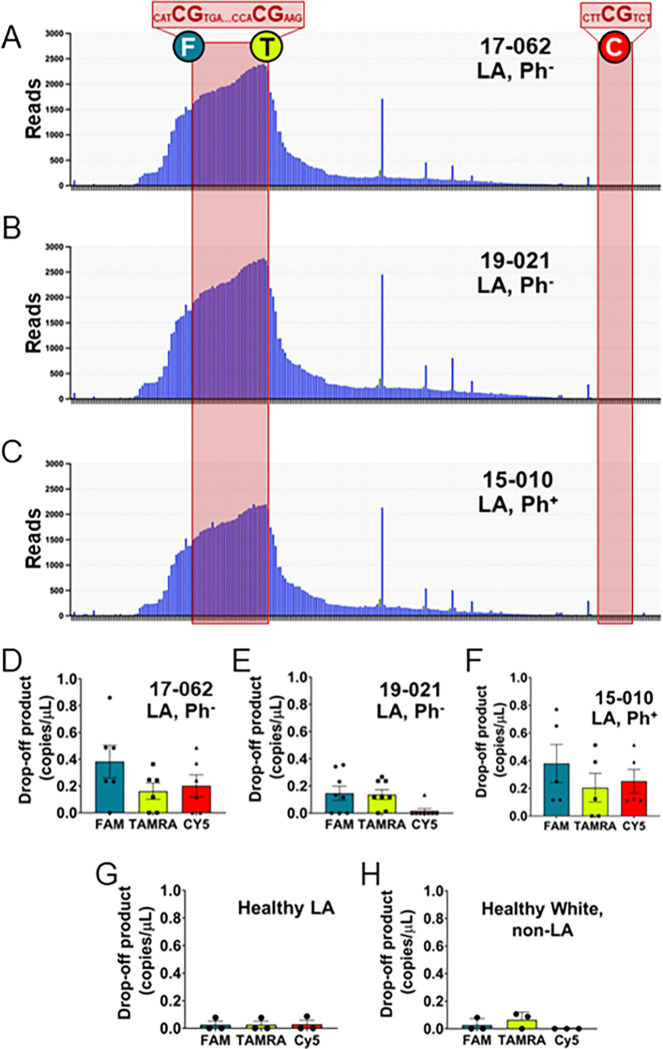
AmpSeq and dPCR of human patient samples shows increased indels at *CRLF2* in ALL patients, but not healthy donors. Indels detected by AmpSeq in patients **(A)** 17–062, **(B)** 19–021, and **(C)** 15–010. The ABC regions with CpG sites are shaded in red. The location of the fluorescent probes used in the subsequent dPCR are indicated by the colored circles containing an “F” (FAM), “T” (TAMRA), or “C” (CY5). Further sequence details are shown in Supplemental Figure 1A. Corresponding dPCR results are indicated for patients **(D)** 17–062, **(E)** 19–021, and **(F)** 15–010. Details of patient samples are indicated in Table 1. dPCR copies/mL indicates mean values ± SEM. dPCR was also performed on gDNA from **(G)** 3 healthy LA donors and **(H)** 3 healthy White, non-LA donors. Quantification of drop-off product is indicated for each site with 3–6 technical replicates done for each patient sample.

To apply our approach to monitor genome instability at ABC sites in LA populations that are susceptible to Ph-like ALL, we leveraged a set of primary samples from the UC Irvine Hematological Malignancies Biorepository, which included two LA patients with Ph-like ALL (17–062 and 19–021) and two LA patients with Ph^+^ ALL (15 – 010 and 17–061) (Table 1) that contained ample cells to provide gDNA for AmpSeq. While the number of samples is small due to the relative rarity of these cases, it is adequate for the exploratory nature of this study as we continue to build our biorepository. Strikingly, for the *CRLF2* ABC, there are remarkable similarities in terms of indels with each LA patient presenting a major peak centered over two CpGs that are proximal to each other (23 bp apart), yet not at a more distal CpG 150 bp away (Figs. 4A–4C, and Supplemental Fig. 4A), regardless of being Ph-like versus Ph^+^.

**Figure 5:**
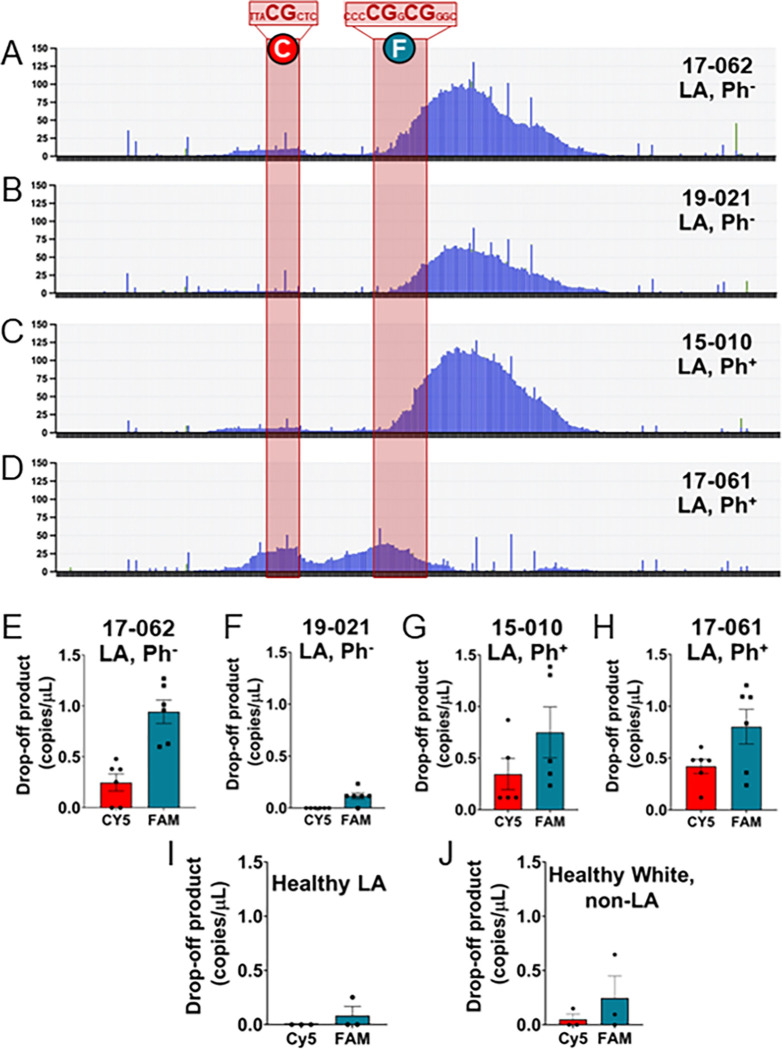
AmpSeq and dPCR of human patient samples shows increased indels at *BCL2* in ALL patients, but not healthy donors. Indels detected by AmpSeq in patients **(A)** 17–062, **(B)** 19–021, **(C)** 15–010, and **(D)**17–061. The ABC regions with CpG sites are shaded in red. The location of the fluorescent probes used in the subsequent dPCR are indicated by the colored circles containing a “C” (CY5) or “F” (FAM). Further sequence details are shown in Supplemental Figure 1A. Corresponding dPCR results are indicated for patients **(E)** 17–062, **(F)** 19–021, **(G)** 15–010, and **(H)** 17–061. Details of patient samples are indicated in Table 1. dPCR copies/mL indicates mean values ± SEM. dPCR was also performed on gDNA from **(I)** 3 healthy LA donors and **(J)** 3 healthy White, non-LA donors. Quantification of drop-off product is indicated for each site with 3–6 technical replicates done for each patient sample.

The *BCL2* ABC showed a more diverse pattern of indel formation in patients (Figs. 5A–5D), similar to what we found in the Nalm6 and Reh cell lines. While translocations involving this region are typically associated with follicular lymphoma, which does not present as a LA health disparity,^14^ the mechanism of DSB formation instigated by AID is thought to be similar,^3^ thus making it important to monitor other ABC sites in LA patients for genome instability. For LA Ph-like patients 17–062 and 19–021 and LA Ph + patient 15 − 010, similar patterns are observed where indels accumulate and peak just after the CpG sites. For patient 17–061, however, we see that the indels form two distinct peaks that line up with the 2nd and 3rd cluster of CpGs (Fig. 5D). As we describe further below, patient 17–061 has other features that suggest a higher level of genome instability. Overall, this indicates that LA patients presenting with increased instability at ABC sites are at higher risk for B cell cancers, making this type of analysis a useful diagnostic tool for cancer risk and severity.

### dPCR of LA patient samples shows increased indels at ABCs

As dPCR uses only a fraction of the DNA used for high-throughput sequencing and provides absolute quantification, there would be a clear advantage to using this method to detect instability at ABC sites. Direct comparison of AmpSeq and dPCR data from gDNA isolated from LA patients with either Ph-like ALL (17–062 and 19–021) or Ph^+^ ALL (15 − 010 and 17–061) also reveals that dPCR has a greater sensitivity to detecting indels. Patient 17–062, for example, shows elevated drop-off at the FAM and TAMRA sites, as shown in the AmpSeq data, yet the dPCR also detects indels at the more distal CpG covered by the Cy5 probe (Fig. 4D). In contrast, patient 19–021 shows no significant accumulation of indels at the Cy5 CpG site, only at the FAM and TAMRA-bound CpG sites despite AmpSeq showing a similar number of reads as 17–062 (Fig. 4E). The dPCR profile for the Ph^+^ ALL patient 15 − 010 (Fig. 4F) also shows elevated drop-off signal at all three sites, including that Cy5 CpG site.

The LA Ph^+^ ALL patient 17–061 is the most distinct and provides a direct example of how AmpSeq can miss crucial sequence information. When attempting to calculate the quantity of drop-off product, which relies on having nearly all amplicons bound by the SUN reference probe, we noted a significant decrease in the total SUN signal that was verified by calculating the total copies/μL based on the amplicon binding of all 4 probes (**Supplemental Figs. 4B and 4C**). Indeed, when we look at the calculation of copies/μL for each probe, we see that the SUN signal is exactly half that of the FAM, TAMRA, and Cy5 probes in each case (**Supplemental Fig. 5B**). This strongly suggests copy number variation (CNV), where this particular PAR sequence is only intact on either the X or Y chromosome in this patient. Relying on AmpSeq data alone would have led us to miss this instability, likely either because an amplicon was not able to be generated due to the sequence anomaly or the variant calling software was unable to align it with the reference and removed it as a low-quality read.

We can also compare the *BCL2* ABC AmpSeq data to what was obtained from dPCR using the drop-off assay designed for this region. For the LA Ph-like patients, 17–062 and 19–021, most indels accumulate immediately after two CpG sites covered by a FAM probe (Figs. 5A and 5B) and we see that FAM drop-off is much higher in patient 17–062 along with increased drop off of the Cy5 probe (Fig. 5E). Patient 19–021 has very little drop-off activity at the *BCL2* ABC with no detectable drop-off of Cy5 and only slightly increased drop-off of FAM (Fig. 5F), again something that is not easily discernable from the AmpSeq data alone. Similar results were also obtained for the LA Ph^+^ patient 15 − 010 (Fig. 5G). The other LA Ph^+^ patient, 17–061, was the only one with a substantial increase in the number of indels at the Cy5-bound CpG site, having higher and more consistent drop-off product at both sites (Fig. 5H). Also, 17–061 does not display evidence of CNV at the *BCL2* locus on chromosome 18. Overall, we see that dPCR can outperform AmpSeq for quantification of indels at ABC sites and can be done in a fraction of the time using between 10–20 ng of gDNA.

While all four of these samples are LA, they are also all cancer patients with some form of ALL. To determine if we detect drop-off from the dPCR assay in healthy individuals, we obtained gDNA from three LA and three White, non-LA donors. Regardless of race, we were unable to detect any significant drop-off product at the *CRLF2* locus from either LA (Fig. 4G) or White, non-LA (Fig. 4H) donors. This was also true when we examined the *BCL2* locus (Figs. 5I and 5J). According to Table 1, blood and bone marrow were collected from ALL patients when their blast count was very high, meaning the B-ALL cells are overrepresented in these patients resulting in measuring increased drop-off products from the cancer cells. In healthy patients, cells in a pre-disease state that are obtained from blood and bone marrow are likely very few. Monitoring of LA individuals at high risk for Ph-like ALL through dPCR could provide an early indicator of disease where there is an increase in B cells with genome instability at ABC sites.

#### Differential expression patterns in Ph-like vs Ph + ALL and between Ph-like ALL cohorts

**Figure 6:**
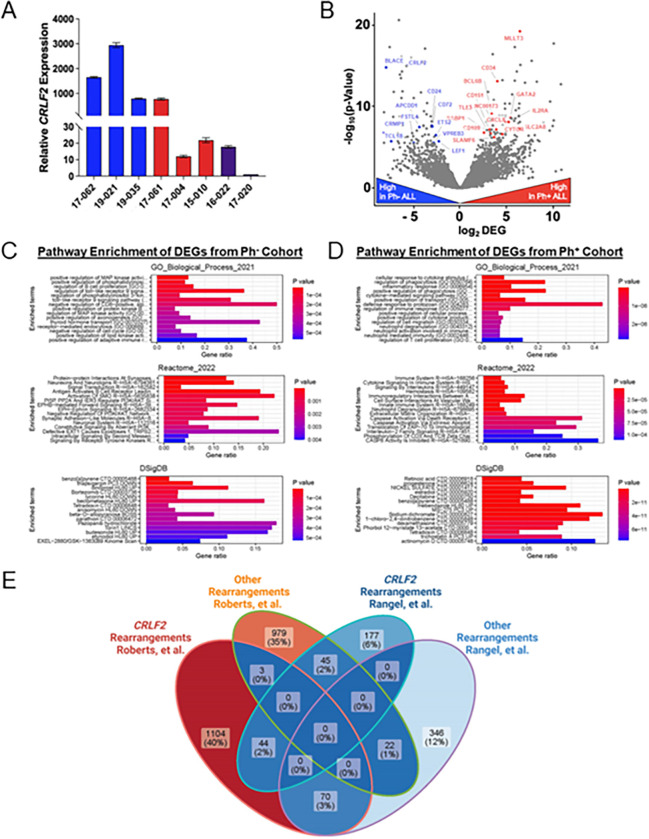
Expression analysis on Ph-like, Ph−, and Ph+ ALL cohorts shows detection of variable *CRLF2* and AID expression levels in LA patients. **(A)** Relative expression of *CRLF2* from the indicated patient samples. Blue are LA Ph-like or Ph−, red are LA Ph+, and purple are Asian Ph+ ALL. **(B)** Volcano plot depicting differently expressed genes (DEGs) in 3 LA Ph^−^ ALL (2 or which are Ph-like) patients (blue), 3 LA Ph^+^ ALL patients (red). A complete list of DEGs has been deposited online (SRA, SPR# accession pending). Gene Set Enrichment Analysis (GSEA) was performed using the pathway databases GO Biological Process, Reactome, and Drug Signatures Database (DSigDB) showing pathway enrichment from the **(C)** Ph^−^ patients 17–062, 19–021, and 19–035 and **(D)** Ph^+^ patients 17–061, 17–004, and 15–010. **(E)** Venn diagram showing overlap of DEGs from this study separated by *CRLF2* vs other rearrangements and Roberts, et al^44^ separated by *CRLF2* vs other rearrangements.

High *CRLF2* expression is a hallmark of Ph-like ALL but given the high genome instability associate with ALL and that *CRLF2* rearrangements can co-occur in Ph + ALL cases,^43^ we wanted to determine *CRLF2* expression levels in all the ALL samples for which we had access. In addition to LA patients, we also included two non-LA Asian Ph + samples (16–022 and 17–020, Table 1) to provide a baseline of expression in a background with very low *CRLF2*-linked Ph-like ALL risk.^19^ All three LA Ph^−^ patients, including the two that were Ph-like, showed high relative expression of *CRLF2* (Fig. 6A). Strikingly, the Ph^+^ patient 17–061, which showed the highest level of genome instability via dPCR, also had a significantly elevated *CRLF2* expression level. This further emphasizes that confirmation of the Philadelphia chromosome should still be followed-up by testing for *CRLF2* overexpression or *CRLF2* rearrangements since standard therapy for Ph^+^ ALL may be less effective in this situation.

Next, to compare global gene expression changes between Ph^−^ and Ph^+^ ALL patients, we preformed RNA-seq comparing Ph-like patients 17–062, 19–021, and Ph− ALL patient 19–035 to Ph^+^ patients 17–061, 17 – 004, and 15 – 010, all of which are LA (Table 1), with analysis of differentially expressed genes (DEG) showing a number of differences in the expression pattern (Fig. 6B). Notably, *CRLF2* expression is significantly higher in all the Ph^−^ samples. As *CRLF2* overexpression is a clinical feature of nearly 65% of all Ph-like ALL cases,^20^ and Ph-like ALL cases are predominantly in LA individuals, it is possible that further testing of patient 19–035 would confirm a Ph-like ALL diagnosis. A complete list of all genes that showed differential expression has been deposited online (SRA, SPR# accession pending).

We further highlight the differences between these two cohorts by performing Gene Set Enrichment Analysis (GSEA) on the Ph^−^ and Ph^+^ datasets to determine pathway enrichment of DEGs using the Gene Ontology Biological Process, Reactome, and Drug Signatures Database (DSigSB) databases. The analysis reveals very little overlap between enrichment in the Ph^−^ (Fig. 6C) and Ph^+^ (Fig. 6D) patients. That several of the most significant pathways affected in Ph^−^ patients include those upregulating the MAP kinase pathway, regulation of B cell proliferation, and negative regulation of cell cycle likely indicate why Ph-like ALL (at least 2/3 of patients) is a much more aggressive disease than Ph^+^ ALL.

While rates of Ph-like ALL among LAs is increasing, it is still a relatively rare disease, making it difficult to collect a large cohort. To further explore the complex heterogeneity of this disease, we compared our RNA-seq data to a set previously published by Roberts, et al.^44^ We intersected significant (P < 0.01) DEGs between the two studies that allowed us to determine overlap between the 15 Ph-like ALL patients from Roberts, et al. with our Ph-like ALL cases as well as the ALL cases with no *CRLF2* rearrangements. Importantly, we are only including the LA cases from this study in our analysis. As strongly suggested by Fig. 6B, even within our own cohort there is 0% overlap between the Ph-like ALL patients and the others (Fig. 6E). Strikingly, only 44 (2%) DEGs overlap between our study’s patients with *CRLF2* rearrangements and the ones from Roberts, et al. Primarily, this demonstrates the complex gene expression patterns in these ALL patients that changes significantly depending on age and ethnicity, the uniqueness of different patient cohorts, and the challenge ahead for defining set characteristics among populations with significant genetic admixture that define Ph-like ALL risk.

## Discussion

**Figure 7:**
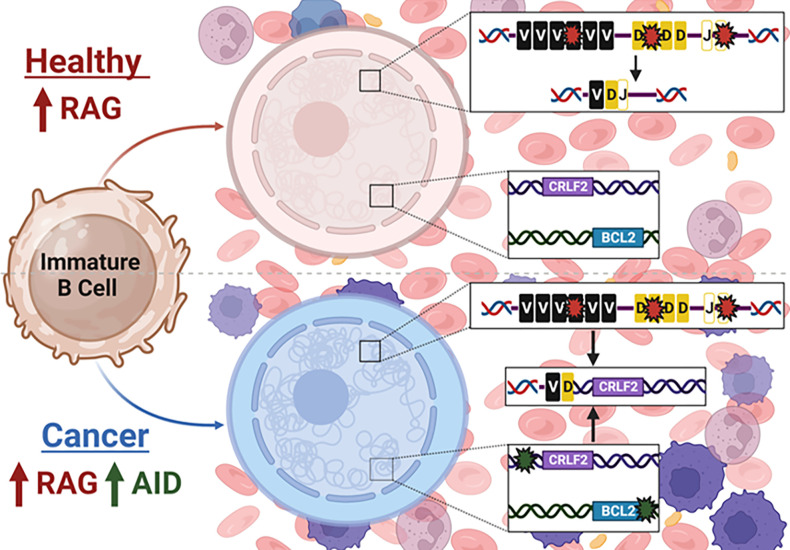
Model of chromosomal translocation formation with the *IGH* locus in B cell cancers. Healthy pro-B/pre-B cells express high levels of the Recombination Activating Gene (RAG) complex to carry out V(D)J recombination to maintain functional adaptive immunity. Cells are transformed into a cancer state when high levels of RAG and AID are present simultaneously to generate DSBs at both *IGH* and ABC sites at *CRLF2* and *BCL2*, among others, leading to oncogenic translocations.

Our major goal is to understand the etiology of several types of B cell malignancies that may appear quite different, but are related by the fact that they carry a chromosomal translocation that occurs at the pro-B/pre-B cell stage of development based on translocation junction analysis.^10,13^ Two such rearrangements are shown in Fig. 1. The *CRLF2-IGH* translocation presents in Ph-like ALL, a disease of immature B cells, and the rearrangement involves a DSB occurring between D and J cassettes of the *IGH* locus that can only consistently occur through the activity of the RAG complex. The *BCL2-IGH* translocation occurs in over 85% of follicular lymphoma and 20% of diffuse large B-cell lymphoma,^45^ which are both diseases of mature B cells. Yet, the translocation junction again shows that the *IGH* DSB was initiated by the RAG complex, thus indicating that the translocation is of pre-B cell origin. B cells harboring this translocation continue to mature and acquire additional mutations until it fully transforms into a mature B cell lymphoma.^46^ This evokes a model where high levels of RAG activity in pre-B cells combined with aberrant AID activity at ABCs generates the two DSBs required for a cancer initiating chromosomal translocation (Fig. 7).

Through our cell-based model, we were able clearly demonstrate a dose response where, as AID levels increase, the ABCs at both *CRLF2* and *BCL2* are subjected to increased genome instability. This was not true for control sites that harbor AID-preferred target sequences but are not known to be involved in oncogenesis. Furthermore, using gDNA from patient samples, we demonstrated that LA patients diagnosed with ALL had significantly higher levels of genome instability at the *CRLF2* and *BCL2* ABCs that was not present in either healthy LA or healthy White, non-LA patients. These results indicate that we may have a clear metric to determine particularly aggressive cancers that have wide-spread mutations due to aberrant AID expression by measuring drop-off at ABC sites in patients diagnosed with ALL. Furthermore, it would provide a more rapid indicator over current standards to allow patients to be more quickly put on an effective treatment regimen.

Diagnosis of Ph-like ALL has remained difficult due to the lack of agreement on what type of gene expression profile or rearrangement constitutes Ph-like ALL, the heterogeneity of mutations and rearrangements in patients, and the lack of concordance regarding rearrangements in the United States versus Europe due to differences in genetic ancestry.^20^ Currently several different assays need to be conducted to confirm a Ph-like ALL diagnosis including chromosomal microarray, next generation sequencing (NGS), flow cytometry for increased *CRLF2* receptor at the cell surface, and FISH analysis of cytogenetically cryptic rearrangements. Despite evidence that AID is involved in the DSB leading to the *CRLF2-IGH* translocation, increased AID activity is not currently examined in any of these diagnostic assays. Likely, this is because increased AID levels occur transiently following exposure to external stimuli, and do not remain high in cells throughout the course of disease. As we demonstrate here, a peak of AID activity in immature B cells can increase mutations and indels at ABCs that will remain in the cells as information scars.

Overall, this analysis fills a critical gap between developing genetic assays in cells to monitor DSB formation and repair via expression of a selectable or detectable marker and NGS of primary or patient material to measure genome instability at a specific locus. In addition to using drop-off assays to examine mutations created at ABC sites as an indicator of instability caused by aberrant AID levels in pre-B cells, allele specific probes can also be designed for mutations associated with cancer subtypes. Ph-like ALL, for example, often has mutations in *JAK2* and *IKZF1*, particularly in cases where a *CRLF2* rearrangement is also present.^20,47^ These additional mutations can indicate a more aggressive cancer that may be responsive to therapies other than the standard of care.^48^

As the cancer field continues to move towards personalized medicine approaches to treatment, it is going to be important to not only take a holistic approach to studying cancer genomes that incorporates multiple races and ethnicities, but also to develop rapid and economical approaches to assessing cancer risk that are widely available to people across socioeconomical stratifications. We are currently lagging in both of these goals. Extensive analysis of a larger cohort of LA patients will allow us to develop diagnostics that can indicate risk for diseases such as Ph-like ALL and determine how factors such as genetic ancestry may be driving cancer health disparities and aid in the discovery of new therapies.
